# PANoptosis links gut dysbiosis to obstructive sleep apnea-associated atherosclerosis: a gut-vascular inflammatory axis

**DOI:** 10.3389/fimmu.2026.1901792

**Published:** 2026-07-09

**Authors:** Ji`an Li, Yunyan Dai, Hong Zhang, Xin Zhou

**Affiliations:** 1 Chengdu University of Traditional Chinese Medicine, Chengdu University of Traditional Chinese Medicine School of Basic Medicine, Chengdu, China; 2 Traditional Chinese Medicine Hospital of Meishan, Affiliated Meishan Hospital of Chengdu University of TCM, Meishan, China

**Keywords:** atherosclerosis, gut microbiota, gut-vascular axis, intermittent hypoxia(IH), obstructive sleep apnea, PANoptosis

## Abstract

Obstructive sleep apnea (OSA) is increasingly recognized as an independent contributor to atherosclerotic cardiovascular disease, yet the mechanisms linking nocturnal intermittent hypoxia (IH) to plaque progression remain incompletely understood. Beyond oxidative stress and sympathetic activation, emerging evidence suggests that the gut may function as a critical relay organ between OSA and vascular injury. IH reshapes the intestinal ecosystem by altering microbial composition, weakening epithelial and mucus barrier integrity, and remodeling microbial metabolites, thereby increasing systemic exposure to microbial ligands and potentially pro-atherogenic metabolic signals, while reducing protective short-chain fatty acids. Trimethylamine N-oxide(TMAO) and imidazole propionate (ImP) are discussed as representative microbiota-derived metabolites implicated in atherosclerosis, although direct evidence linking OSA-related IH to increased circulating TMAO in human cohorts remains limited. These intestinal inflammatory and metabolic inputs may amplify endothelial dysfunction, macrophage activation, and chronic vascular inflammation. We propose that PANoptosis, an integrated inflammatory cell death program involving pyroptosis, apoptosis, and necroptosis, may represent a plausible downstream inflammatory cell death mechanism through which dysbiosis-associated and plaque-local stressors converge to promote plaque injury. This review summarizes convergent indirect evidence supporting a gut dysbiosis-PANoptosis-vascular injury hypothesis, and discusses potential therapeutic implications for OSA-associated atherosclerosis.

## 1 Introduction

Obstructive sleep apnea (OSA) is a highly prevalent chronic disorder characterized by recurrent upper airway collapse during sleep, leading to cyclical oxyhemoglobin desaturation, reoxygenation, sleep fragmentation, hypercapnia, and sustained cardiometabolic stress (1). Beyond its respiratory manifestations, OSA is now increasingly recognized as a systemic disease with substantial cardiovascular consequences, including hypertension, coronary artery disease, heart failure, stroke, and atherosclerotic vascular injury (2).

Atherosclerosis is the pathological basis of most ischemic cardiovascular and cerebrovascular events and is now recognized as a chronic inflammatory disease involving endothelial dysfunction, lipid deposition, immune cell recruitment, vascular smooth muscle cell remodeling, necrotic core formation, and fibrous cap destabilization (3, 4). Clinical imaging studies further indicate that OSA is independently associated with increased carotid and coronary plaque burden after adjustment for obesity and other conventional risk factors, supporting a direct link between sleep-disordered breathing and accelerated atherogenesis (5).

Intermittent hypoxia(IH), the defining pathophysiological hallmark of OSA, is widely considered the most biologically damaging component of the syndrome (6, 7). Repetitive hypoxia-reoxygenation cycles induced by IH promote reactive oxygen species(ROS) generation, inflammatory pathway activation, endothelial injury, and vascular remodeling (8, 9). Although continuous positive airway pressure therapy may attenuate part of this inflammatory and oxidative burden, residual cardiovascular risk often persists, suggesting that additional pathogenic mechanisms remain active in OSA (6). The gut microbiota has emerged as a central regulator of host metabolism and immune homeostasis, with growing evidence linking dysbiosis to OSA-related cardiovascular injury (10). In both clinical OSA cohorts and experimental IH models, hypoxic stress alters microbial community structure, reduces short-chain fatty acid-producing commensals, perturbs microbial metabolite profiles, and impairs intestinal barrier integrity (11–13). These findings suggest that microbiota-derived inflammatory signals and metabolites may influence vascular homeostasis and plaque biology through the gut-vascular axis (14, 15). Supporting studies combining IH with hypercapnia or metabolic stress have shown coordinated alterations in the gut microbiome, metabolome, and atherosclerotic phenotype (16, 17).

**Figure 1:**
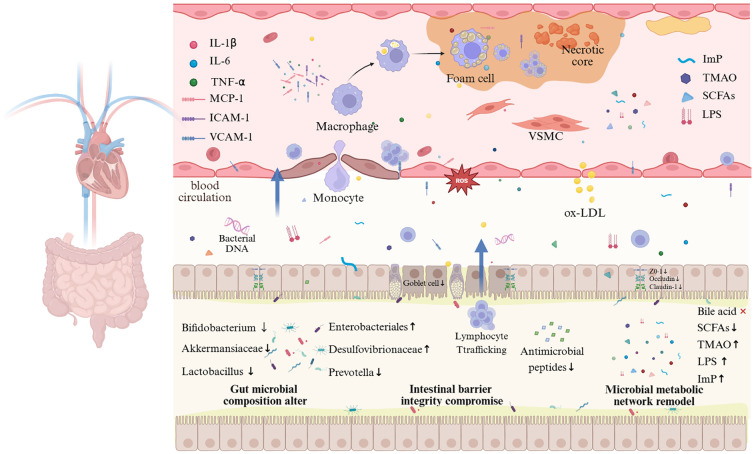
Intermittent hypoxia-driven gut dysbiosis and the gut-vascular axis in OSA-associated atherosclerosis. Recurrent intermittent hypoxia (IH) in obstructive sleep apnea (OSA) reshapes the intestinal ecosystem by altering microbial composition, weakening epithelial and mucus barrier integrity, and remodeling microbial metabolite output. These changes may promote loss of short-chain fatty acid (SCFA)-producing commensals, enrichment of pro-inflammatory pathobionts, increased gut permeability, and systemic translocation of microbe-associated molecular patterns (MAMPs), damage-associated molecular patterns (DAMPs), and microbial metabolites. TMAO and imidazole propionate (ImP) are shown as representative pro-atherogenic microbial metabolites, although direct evidence linking OSA-related IH to increased circulating TMAO in human cohorts remains limited. Reduced SCFA availability may further impair epithelial barrier repair and immune homeostasis. The resulting gut-derived inflammatory and metabolic spillover may contribute to endothelial dysfunction, monocyte recruitment, macrophage activation, foam cell formation, and plaque progression. Established vascular inflammation may in turn aggravate intestinal dysbiosis and barrier dysfunction, forming a self-amplifying gut-vascular loop. Created with Biorender.com.

However, one critical mechanistic question remains unresolved: how are diverse upstream insults translated into inflammatory cellular injury within the atherosclerotic plaque? PANoptosis, a recently described inflammatory programmed cell death modality integrating pyroptosis, apoptosis, and necroptosis through PANoptosome assembly, may offer a plausible candidate mechanism (18, 19). In plaques exposed to IH-derived oxidative stress, microbial ligands, and oxidized lipids, this integrated form of cell death may be more biologically relevant than single-pathway models. This review therefore proposes a gut-vascular inflammatory framework in which IH-induced gut dysbiosis may contribute to atherosclerotic plaque injury, while PANoptosis may be a testable downstream mechanistic hypothesis underlying atherosclerotic plaque injury and instability (**Figure 1**).

## 2 IH reshapes the gut ecosystem in OSA

IH is not only a respiratory and vascular stressor but also a strong ecological pressure on the intestinal microenvironment. Through repeated hypoxia-reoxygenation cycles, IH promotes oxidative stress, inflammatory signaling, epithelial injury, and microbial metabolic remodeling, thereby converting the gut from a homeostatic barrier organ into a systemic source of inflammatory and metabolic cues (20, 21). In OSA-associated atherosclerosis, three IH-driven changes are especially relevant to gut ecosystem.

### 2.1 IH alters gut microbial composition

Hypoxic injury can impair intestinal function and alter the composition and metabolism of gut microbiota (22). Animal and clinical studies indicate that IH reshapes gut microbial communities, although the exact taxonomic changes vary with species, diet, exposure duration, and metabolic background. Overall, IH-related dysbiosis is characterized by loss of beneficial commensals and enrichment of bacteria associated with inflammation or cardiometabolic risk (23). These effects may be magnified by obesity and metabolic syndrome, two conditions that frequently coexist with OSA. Clinical observations support a close relationship between nocturnal hypoxic burden and gut dysbiosis. Patients with OSA show altered gut microbial composition and reduced abundance of short-chain fatty acids(SCFAs)-producing taxa, suggesting impaired microbial capacity to sustain epithelial and immune homeostasis (11). Importantly, microbial changes appear to correlate more closely with cumulative hypoxic burden than with apnea frequency alone, implying that oxygen desaturation metrics may better reflect biological stress on the gut ecosystem (12). These findings support the view that IH acts as a selective ecological driver of a pro-inflammatory and metabolically unfavorable intestinal configuration.

### 2.2 IH compromises intestinal barrier integrity

Barrier dysfunction is a critical step that converts local dysbiosis into systemic vascular risk. The intestinal barrier consists of epithelial tight junctions(TJ), the mucus layer, antimicrobial factors, immune cells, and local vascular interfaces (24). IH disrupts this multilayer structure through oxidative stress, inflammatory activation, and epithelial metabolic injury. Repetitive hypoxia-reoxygenation upregulates ROS generation, a central mechanism linking OSA to cardiovascular endothelial damage (20). IH has also been shown to aggravate barrier disruption through cAMP/PKA/RhoA-related cytoskeletal remodeling, supporting the broader principle that hypoxic stress destabilizes barrier systems across tissues (25). At the intestinal level, IH-associated oxidative and inflammatory stress may reduce TJ integrity and increase permeability. Altered gut epithelial barrier markers have been documented in patients with OSA, and systematic evidence supports the presence of barrier dysfunction in this setting (26, 27). In experimental models, chronic intermittent hypoxia impairs intestinal barrier function, whereas melatonin can attenuate this injury, suggesting roles for oxidative stress and circadian-metabolic disruption (28).The mucus layer provides an additional protective interface that limits direct microbial contact with epithelial cells. Goblet cell-derived mucins, particularly mucin 2, are essential for intestinal homeostasis. IH interferes with the differentiation and secretory functions of goblet cells, leading to a reduction in mucus layer thickness, particularly compromising the integrity of the inner sterile mucus layer (29). Although direct adult OSA evidence remains limited, recurrent IH is biologically likely to weaken mucus barrier defense and facilitate microbial-epithelial interaction.

### 2.3 IH remodels the microbial metabolic network

The vascular significance of IH-induced dysbiosis depends not only on microbial composition but also on altered microbial function. Reduced SCFAs-producing capacity may remove an important anti-inflammatory and barrier-protective input, whereas enrichment of microbial pathways related to pro-atherogenic metabolites may promote vascular injury (11, 30). SCFAs help sustain epithelial metabolism, immune tolerance, and endothelial homeostasis; their depletion lowers the threshold for systemic inflammation (31, 32).

Trimethylamine N-oxide(TMAO) is the best-characterized pro-atherogenic microbial metabolite in atherosclerosis. Gut microbes convert dietary choline, phosphatidylcholine, and carnitine into trimethylamine(TMA), which is subsequently oxidized in the liver. Foundational research established this pathway as a major microbiota-host mechanism in atherosclerosis (33), and subsequent studies linked TMAO to inflammation and cardiovascular disease severity (34–37). In OSA, however, direct evidence that IH consistently increases circulating TMAO in human cohorts remains limited. Thus, the involvement of TMAO in OSA-associated atherosclerosis should be interpreted mainly as an inference based on IH-related dysbiosis, altered microbial metabolic potential, and experimental models combining IH or intermittent hypoxia/hypercapnia with atherosclerotic susceptibility (16, 17). Imidazole propionate(ImP) has also been implicated in cardiometabolic disease and more recently identified as a driver and therapeutic target in atherosclerosis (38, 39). Accordingly, IH-induced dysbiosis should be viewed as a functional metabolic reprogramming event rather than a simple taxonomic disturbance.

## 3 Gut microbiota dysbiosis and atherosclerosis: a bidirectional inflammatory loop

Gut microbiota dysbiosis and atherosclerosis are increasingly viewed as interconnected components of a bidirectional gut–vascular inflammatory loop. Dysbiosis may contribute to plaque development through inflammatory, metabolic, and barrier-related mechanisms, including enrichment of pro-inflammatory pathobionts, depletion of barrier-protective commensals, altered bile acid and SCFAs metabolism, increased gut permeability, and enhanced systemic immune activation (40). Conversely, established atherosclerosis and its associated dyslipidemia, oxidative stress, and systemic inflammation may further reshape the intestinal microenvironment and reinforce dysbiosis.

### 3.1 Dysbiosis may promote vascular inflammation and plaque progression

The primary mechanism linking dysbiosis to atherosclerosis is microbial translocation. When barrier integrity is damaged, MAMPs such as lipopolysaccharide(LPS), peptidoglycan, and bacterial DNA enter the circulation and activate innate immune signaling in endothelial cells, monocytes, and macrophages. This promotes adhesion molecule expression, cytokine production, monocyte recruitment, and foam cell formation-key pathological features in plaque development (41). Increased gut permeability may also facilitate pathogenic interactions between microbial products and oxidized lipoproteins (42). The second mechanism involves the migration of immune cells from the gut to the periphery. In an obesity-inducing dietary model, microbiota dysbiosis promotes the migration of intestinal lymphocytes into the circulation and exacerbates atherosclerosis (43). This observation is highly relevant to OSA, where IH, obesity, and metabolic inflammation often coexist. The third mechanism is microbial metabolic reprogramming. TMAO, bile acid derivatives, ImP, and SCFAs have emerged as central mediators of microbiota-vascular communication (44, 45). Bile acids are particularly important because they connect gut microbial metabolism to cholesterol homeostasis and inflammatory signaling through receptors such as FXR and TGR5 (46). Conversely, dietary fiber-derived microbial products can be protective. For example, pectin supplementation attenuated atherosclerosis by promoting Akkermansia-related acetic acid metabolism (47). Genetic and experimental evidence also supports causality: Mendelian randomization analyses suggest causal associations between specific gut microbial taxa and atherosclerosis (48).

### 3.2 Atherosclerosis may reinforce gut dysbiosis and intestinal inflammation

The relationship of the intestinal microenvironment and atherosclerotic vascular microenvironment is bidirectional. Established atherosclerosis is accompanied by systemic inflammation, dyslipidemia, oxidative stress, and altered bile acid signaling, all of which can reshape the intestinal microenvironment and further impair barrier function (41, 47). This may help explain why gut dysfunction and vascular inflammation frequently coexist in cardiometabolic disease. Intestinal fungal dysbiosis has also been implicated: Candida albicans accelerates atherosclerosis by activating intestinal hypoxia-inducible factor-2α signaling (49). Increased gut permeability and oxidized low-density lipoprotein(ox-LDL) have been associated with systemic inflammatory conditions, suggesting that barrier dysfunction and lipid oxidation can sustain one another (42). Therapeutic studies reinforce the functional importance of this bidirectional loop. Berberine can reduce atherosclerosis partly by downregulating the choline-TMA-TMAO pathway in gut microbiota, while its broader vascular benefits may involve combined effects on inflammation and host metabolism (50, 51).

Overall, gut dysbiosis and atherosclerosis may reinforce each other within a bidirectional gut-vascular inflammatory loop. This framing also clarifies that gut-derived signals are unlikely to be the sole drivers of plaque inflammatory cell death. In this review, we emphasize the gut-to-vascular direction because IH, the defining stressor in OSA, can alter microbial composition, barrier integrity, and microbial metabolic output before or alongside vascular injury. Thus, microbial ligands and metabolites may act as upstream licensing signals, whereas established vascular inflammation, oxidative stress, dyslipidemia, and cytokine-rich plaque microenvironments may amplify PANoptosis-related pathways independently or synergistically. PANoptosis is therefore proposed as a convergent mechanism within this bidirectional network rather than as a process driven exclusively by the gut.

## 4 PANoptosis as a proposed bridge between intestinal inflammation and vascular damage

The gut-vascular axis explains how IH-induced dysbiosis generates systemic vascular stress, but it does not fully clarify how these upstream inputs converge with local plaque injury to produce destructive cellular outcomes. PANoptosis, an integrated inflammatory cell death program coordinated by PANoptosome complexes containing context-dependent sensors, adaptors, and effectors (52–54), may fill this mechanistic gap. However, direct evidence demonstrating PANoptosome assembly in plaques from OSA patients or IH-exposed atherosclerotic models is currently lacking. Therefore, PANoptosis is discussed here as a testable hypothesis supported by convergent indirect evidence.

### 4.1 PANoptosis integrates inflammatory death pathways

PANoptosis emerged from the recognition that programmed cell death pathways are not independent modules but highly interconnected inflammatory networks. TNF-α plus caspase inhibition can induce necrotic-like cell death (55), while RIPK1, RIPK3, MLKL, caspase-8, and inflammasome-related molecules coordinate the balance among apoptosis, necroptosis, and pyroptosis (56–59). Gasdermin pores can further amplify apoptotic signaling by permeabilizing mitochondria, illustrating direct crosstalk between lytic and non-lytic death programs (60).

Accordingly, PANoptosis is best understood as a stimulus-dependent cell death network coordinated by PANoptosome complexes rather than as a linear pathway. Depending on the initiating signal, ZBP1, AIM2, NLRP3, RIPK1, ASC, FADD, caspases, RIPK3, MLKL, and GSDMD may participate in different PANoptosome platforms (61–63). Although NLRP12-related PANoptosome formation has been described in other inflammatory settings (64, 65), current evidence is insufficient to support its involvement in OSA-associated atherosclerosis. These properties make PANoptosis particularly relevant to OSA-associated plaques, where microbial ligands, oxidized lipids, mitochondrial stress, cytokines, and hypoxia-reoxygenation injury coexist.

### 4.2 Microbial ligands, metabolic stress, and DAMPs may license PANoptosis-related inflammatory death

We propose a two-hit model in which intestinal inputs and plaque-local stressors converge to increase susceptibility to PANoptosis-related inflammatory death. IH-induced barrier dysfunction may increase systemic exposure to MAMPs and metabolism-associated molecular patterns, including LPS, bacterial DNA, peptidoglycan fragments, and microbial metabolites (66–68). These signals may prime TLRs, NOD-like receptors, interferon-related pathways, and inflammatory transcriptional programs in vascular cells, but should be viewed as licensing rather than exclusive triggering signals.

The second set of signals originates from the plaque microenvironment, where IH and plaque-specific stressors generate endogenous DAMPs, including ROS, ox-LDL, mitochondrial DNA, mitochondrial double-stranded RNA, cholesterol crystal-related injury, and inflammatory cytokines (69–72). These local signals may cooperate with intestinal inputs to promote inflammatory cell death. ZBP1 may respond to nucleic acid stress and interferon signaling; AIM2 may sense cytosolic DNA; NLRP3 may integrate ROS, ion flux, cholesterol crystal injury, and mitochondrial dysfunction; and RIPK1 may scaffold inflammatory death complexes under TNF-α-rich and innate immune-primed conditions (73–75). TMAO may represent a plausible metabolic input into this death-prone state, but its role in OSA-related plaques remains inferential rather than directly proven.

### 4.3 Macrophage and endothelial PANoptosis in plaque progression

Macrophages are likely to be major executors of PANoptotic conversion. Within plaques, they are simultaneously exposed to ox-LDL, cholesterol crystals, gut-derived ligands, TMAO, cytokines, ROS, and defective efferocytosis. This favors integrated inflammatory death rather than a single isolated pathway. RIPK1 expression is associated with inflammation in early human atherosclerosis, and silencing RIPK1 reduces NF-κB activation and atherogenesis in mice (76). Necroptosis-related pathways have been implicated in atherosclerotic macrophage death and have been proposed as therapeutic and diagnostic targets (77, 78). Pyroptotic machinery is also highly relevant. Studies have shown that macrophage-derived GSDMD promotes atherosclerosis through mitochondrial damage and STING-IRF3/NF-κB activation, whereas microbiota-derived butyrate signaling can suppress macrophage pyroptosis (79–81). TMAO further aggravates atherosclerosis through a CD36-dependent MAPK/JNK pathway (82). In plaques with defective efferocytosis, inflammatory macrophage death expands the necrotic core and sustains cytokine release (83).

Endothelial cells provide the interface between circulating gut-derived signals and the arterial wall. RIPK1 inhibition protects against ox-LDL induced endothelial injury (84). Endothelial inflammatory death could increase permeability, DAMPs release, leukocyte adhesion, and lipid entry into the intima. In addition, endothelial-to-mesenchymal transition (EndMT) is common in atherosclerotic lesions and associated with plaque instability (85), while inflammation promotes EndMT and vascular calcification through BMPR2 downregulation (86). It is known that BMPR2 deficiency induces mitochondrial-mediated apoptosis (involving Caspase 9 and Caspase 3) and Caspase 1-driven pyroptosis, accompanied by exacerbated inflammation (87, 88). Acetate can regulate EndMT (89), suggesting that microbial metabolic changes may influence both endothelial phenotype and death susceptibility.

**Figure 2:**
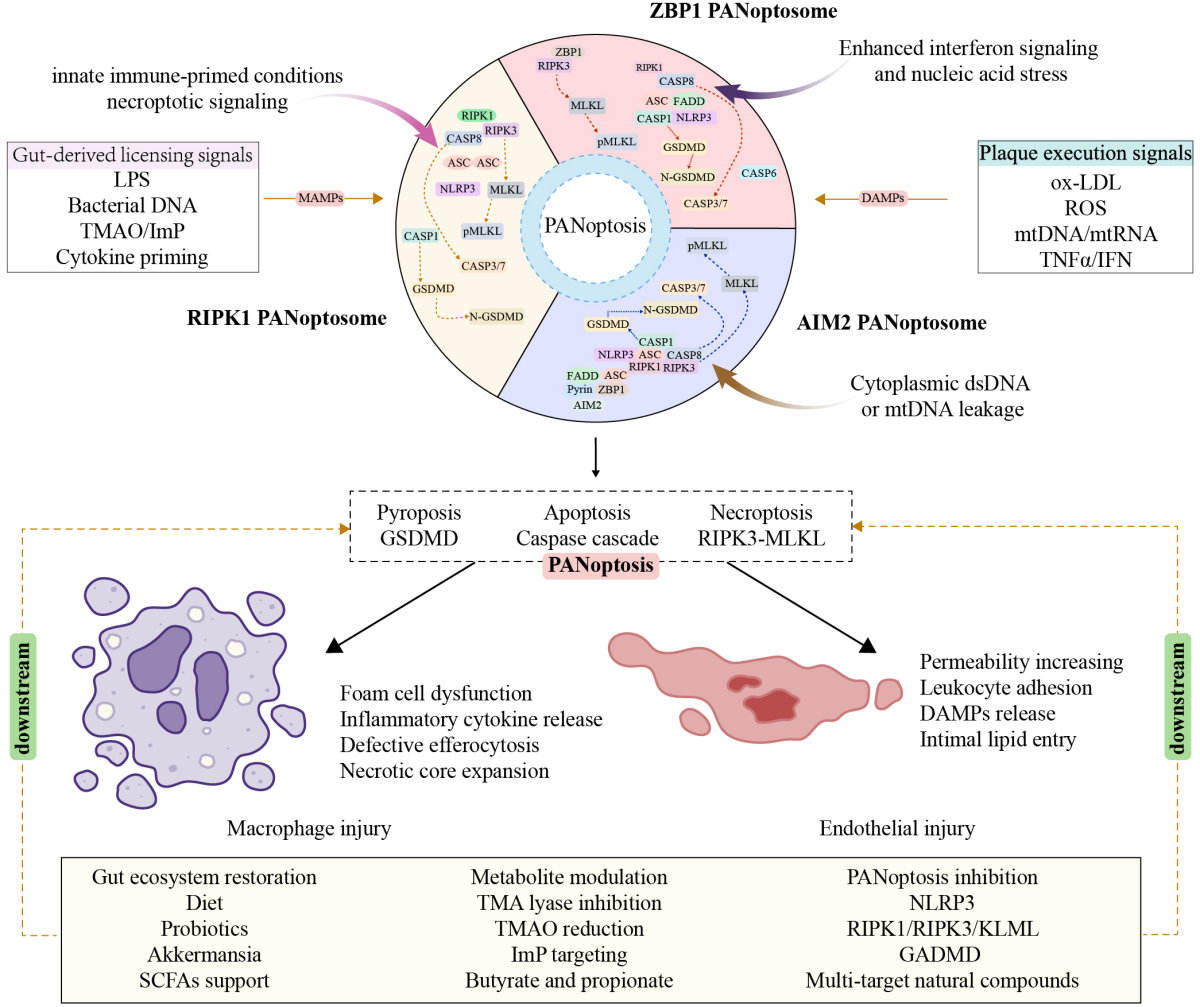
PANoptosis as a proposed intracellular mechanism linking dysbiosis-associated stress to plaque injury. Intestinally derived vascular stressors generated by IH-induced dysbiosis may provide a licensing signal for vascular susceptibility, whereas plaque-local oxidative stress, mitochondrial injury, oxidized lipids, cholesterol crystals, and cytokine-rich inflammation may provide execution signals for PANoptosome activation. Candidate PANoptotic sensors and signaling nodes include ZBP1, AIM2, NLRP3, RIPK1, caspase-8, RIPK3, MLKL, and GSDMD. NLRP12 is omitted in this proposed OSA-atherosclerosis model because no direct evidence supports its involvement. Through coordinated activation of pyroptosis, apoptosis, and necroptosis, PANoptosis-related inflammatory death in macrophages and endothelial cells may promote foam cell dysfunction, inflammatory cytokine release, defective efferocytosis, endothelial barrier injury, leukocyte adhesion, necrotic core expansion, and plaque instability. This hypothetical model reveals future research potential at three levels of the gut-vascular-PANoptosis axis: restoration of the gut ecosystem, modulation of microbial metabolites, and inhibition of PANoptosis-related inflammatory cell death pathways.

Overall, PANoptosis provides a plausible conceptual framework for explaining how intestinally derived vascular stressors and plaque-local injury signals may converge to promote macrophage dysfunction, endothelial damage, necrotic core expansion, and plaque instability. However, direct demonstration of PANoptosome assembly, cell type-specific PANoptosis markers, and causal links between IH-induced dysbiosis and plaque PANoptosis will be required to validate this hypothesis (**Figure 2**).

## 5 Therapeutic implications: targeting the gut-vascular-PANoptosis axis

### 5.1 Restoring gut microbial homeostasis

Because IH alters gut microbial diversity and metabolic output, microbiota-directed interventions represent a rational upstream strategy (90, 91). Diet is likely the most scalable clinical approach. Fiber-enriched and microbiota-modulating dietary patterns may increase protective metabolites, reduce pro-atherogenic microbial products, and support barrier function (92, 93). Probiotic or commensal-based approaches may provide additional benefit, as experimental studies suggest that Enterococcus faecium, Lactobacillus johnsonii, and Akkermansia muciniphila can improve SCFAs-related microbial homeostasis and suppress inflammatory macrophage polarization (94–96). Although these findings remain largely preclinical, they support gut ecosystem restoration as an upstream intervention point in OSA-associated vascular injury.

### 5.2 Modulating microbial metabolites

Regulating metabolite levels may be a more precise medical strategy than targeting microbial composition alone. TMAO is the most established example, linking gut microbial metabolism to bile acid homeostasis, lipid handling, inflammation, and atherosclerotic progression (97). Non-lethal inhibition of microbial TMA production reduces TMAO generation and atherosclerosis in experimental models, providing proof of concept for this strategy (98). However, given the limited direct evidence linking OSA-related IH to increased circulating TMAO in human cohorts, TMAO-targeted strategies remain mechanistically plausible rather than clinically established in OSA-associated atherosclerosis.

Natural compounds such as berberine, resveratrol, and curcumin can attenuate atherosclerotic injury by remodeling gut microbial metabolism and suppressing the TMA-TMAO axis (99–101). However, most evidence comes from preclinical or mechanistic studies rather than OSA-specific clinical trials. Their clinical translation is also limited by poor oral bioavailability, extensive metabolism, formulation variability, and uncertain target exposure. Thus, these agents should be viewed as experimental or adjunctive candidates rather than established therapies. In contrast, restoring beneficial metabolites such as butyrate and propionate may reduce vascular inflammation and improve intestinal cholesterol handling (102–104). Emerging metabolites, including ImP, may further expand this therapeutic landscape (105).

### 5.3 Targeting PANoptosis and related inflammatory death programs

At the downstream level, PANoptosis-related signaling nodes represent candidate therapeutic targets. Transcriptomic studies support the relevance of PANoptosis-related molecular signatures in atherosclerosis (106), and recent reviews have proposed PANoptosis as a therapeutic target in atherosclerotic cardiovascular disease (107). Although no PANoptosis-specific therapy has entered clinical practice, several components of the network are potentially druggable. NLRP3 inhibition can attenuate inflammasome assembly in macrophages (108), while IRF1 contributes to activation of multiple PANoptosome platforms (109). Necroptosis-related targets are also supported by atherosclerosis data: RIP3-mediated macrophage necrosis promotes plaque development, MLKL deficiency reduces necrotic core formation, and miR-223-3p limits macrophage necroptotic death by targeting RIPK3 (110–112). GSDMD-dependent pyroptosis is another promising target, and emodin suppresses NLRP3/GSDMD-related inflammation in atherosclerosis models (113, 114). These observations suggest that selective modulation of inflammatory death pathways may be more appropriate than complete pathway suppression.

### 5.4 Multi-target strategies

Because OSA-associated atherosclerosis involves converging hypoxic, microbial, metabolic, and inflammatory stresses, multi-target interventions may be particularly valuable. Traditional Chinese medicine-derived compounds and natural products often act across several pathogenic layers, including microbial composition, metabolite production, oxidative stress, inflammatory signaling, and programmed cell death (115). Berberine, resveratrol, curcumin, emodin, and apigenin illustrate this network-level potential by acting on the TMA–TMAO axis, endothelial inflammatory responses, or NLRP3/GSDMD-related lytic cell death, mainly in cell and animal models (50, 51, 99–101, 114, 116). However, limited bioavailability, dose-dependent effects, formulation variability, and insufficient OSA-specific clinical evidence remain major barriers. These compounds should therefore be viewed as candidate modulators of the gut-vascular inflammatory network rather than validated anti-PANoptosis therapies.

## 6 Conclusion and future perspectives

OSA-associated atherosclerosis is unlikely to be driven solely by hypoxia, inflammation, or lipid abnormalities. Instead, it may reflect a pathological network involving IH-induced gut dysbiosis, intestinal barrier disruption, microbial metabolic remodeling, systemic immune activation, and inflammatory cell death within the vascular wall. Within this process, the gut-vascular axis helps explain how nocturnal hypoxic stress may be transmitted to the vascular microenvironment, while PANoptosis is proposed as a testable mechanistic downstream hypothesis arising from the convergence of multiple intestinal and plaque-local stress signals.

Although direct clinical evidence linking PANoptosis to OSA-associated atherosclerosis is still lacking. Current studies on IH, gut microbiota, MAMPs, microbial metabolites, NLRP3, RIPK1, GSDMD, endothelial dysfunction, and macrophage inflammatory death collectively support this mechanistic hypothesis. Gut-derived MAMPs and metabolites may provide a licensing signal, whereas oxidized lipids, mitochondrial nucleic acid stress, ROS, and inflammatory cytokines may provide local execution signals. This model integrates IH-driven oxidative stress, microbial translocation, metabolite imbalance, macrophage inflammatory death, endothelial injury, necrotic core expansion, and plaque instability within a testable gut-vascular-cell death framework.

Future studies should determine whether microbial signatures, metabolite profiles, barrier markers, and PANoptosis-related markers correlate with vascular burden and cardiovascular outcomes in OSA. Experimental work should identify susceptible plaque cell types and test whether gut-directed therapy, metabolite modulation, or inhibition of PANoptosis-related pathways provides vascular protection beyond standard OSA management. Sex should also be incorporated as a biological variable, because sex-based differences in OSA phenotype, hypoxic burden, gut microbiome composition, microbial metabolites, and immune-inflammatory responses may modify this axist. Overall, the gut-vascular-PANoptosis axis may provide a coherent and testable framework for understanding residual cardiovascular risk in OSA and may open new translational opportunities toward prevention and treatment for OSA-associated atherosclerosis.
